# Fluid-structure interaction simulation of mechanical aortic valves: a narrative review exploring its role in total product life cycle

**DOI:** 10.3389/fmedt.2024.1399729

**Published:** 2024-07-01

**Authors:** Mariachiara Arminio, Dario Carbonaro, Umberto Morbiducci, Diego Gallo, Claudio Chiastra

**Affiliations:** PoliTo^BIO^Med Lab, Department of Mechanical and Aerospace Engineering, Politecnico di Torino, Turin, Italy

**Keywords:** heart valve prosthesis, mechanical aortic valve, valve fluid dynamics, *in silico* medicine, computer modelling, fluid-structure interaction simulation, credibility assessment, preclinical tests

## Abstract

Over the last years computer modelling and simulation has emerged as an effective tool to support the total product life cycle of cardiovascular devices, particularly in the device preclinical evaluation and post-market assessment. Computational modelling is particularly relevant for heart valve prostheses, which require an extensive assessment of their hydrodynamic performance and of risks of hemolysis and thromboembolic complications associated with mechanically-induced blood damage. These biomechanical aspects are typically evaluated through a fluid-structure interaction (FSI) approach, which enables valve fluid dynamics evaluation accounting for leaflets movement. In this context, the present narrative review focuses on the computational modelling of bileaflet mechanical aortic valves through FSI approach, aiming to foster and guide the use of simulations in device total product life cycle. The state of the art of FSI simulation of heart valve prostheses is reviewed to highlight the variety of modelling strategies adopted in the literature. Furthermore, the integration of FSI simulations in the total product life cycle of bileaflet aortic valves is discussed, with particular emphasis on the role of simulations in complementing and potentially replacing the experimental tests suggested by international standards. Simulations credibility assessment is also discussed in the light of recently published guidelines, thus paving the way for a broader inclusion of *in silico* evidence in regulatory submissions. The present narrative review highlights that FSI simulations can be successfully framed within the total product life cycle of bileaflet mechanical aortic valves, emphasizing that credible *in silico* models evaluating the performance of implantable devices can (at least) partially replace preclinical *in vitro* experimentation and support post-market biomechanical evaluation, leading to a reduction in both time and cost required for device development.

## 1 Introduction

In recent years, there has been a growing interest in applying computational modelling to advance the development of medical devices (1, 2), particularly in the cardiovascular field (3–7). Computational modelling and simulation (CM&S) has emerged as a powerful resource for increasing the efficiency of the total product life cycle of medical devices, including design ideation, device evaluation, safety and efficacy assessment, and post-market modifications (1). Notably, CM&S plays a key role in the pre-clinical phase of medical device development, when the device is designed and optimized before clinical testing. Evidence collected through CM&S in the pre-clinical evaluation phase can support regulatory approval applications, complementing and partially substituting experimental testing (2, 8). In this context, computational models are used to characterize the performance of medical devices with the aim of either reproducing experimental tests or providing results that are difficult and expensive to achieve experimentally (1, 2, 8, 9).

International regulatory bodies are increasingly recognizing the significance of CM&S results in the certification procedures for medical devices. The Food and Drug Administration (FDA) 2022 report on successes and opportunities of CM&S (9) highlighted that computational models are currently considered by the Center for Devices and Radiological Health of FDA for premarket product reviews and post-market product assessment. To be accepted at the regulatory level, *in silico* evidence must be collected through reliable CM&S (8, 10). Therefore, various international associations in the medical device field have elaborated guidelines establishing the requirements that CM&S should satisfy to prove its reliability and its consequent predictive capability in regulatory evaluation processes. In particular, the FDA has recently published a guidance on the credibility assessment of CM&S in medical device submissions, providing recommendations on how to plan and report credibility assessment activities to support the acceptance of CM&S results (11). This guidance proposes a risk-based credibility assessment framework, integrating the framework presented by the American Society of Mechanical Engineers (ASME) in a previous standard concerning the verification and validation of CM&S for medical devices (ASME V&V 40-2018) (12).

**Figure 1:**
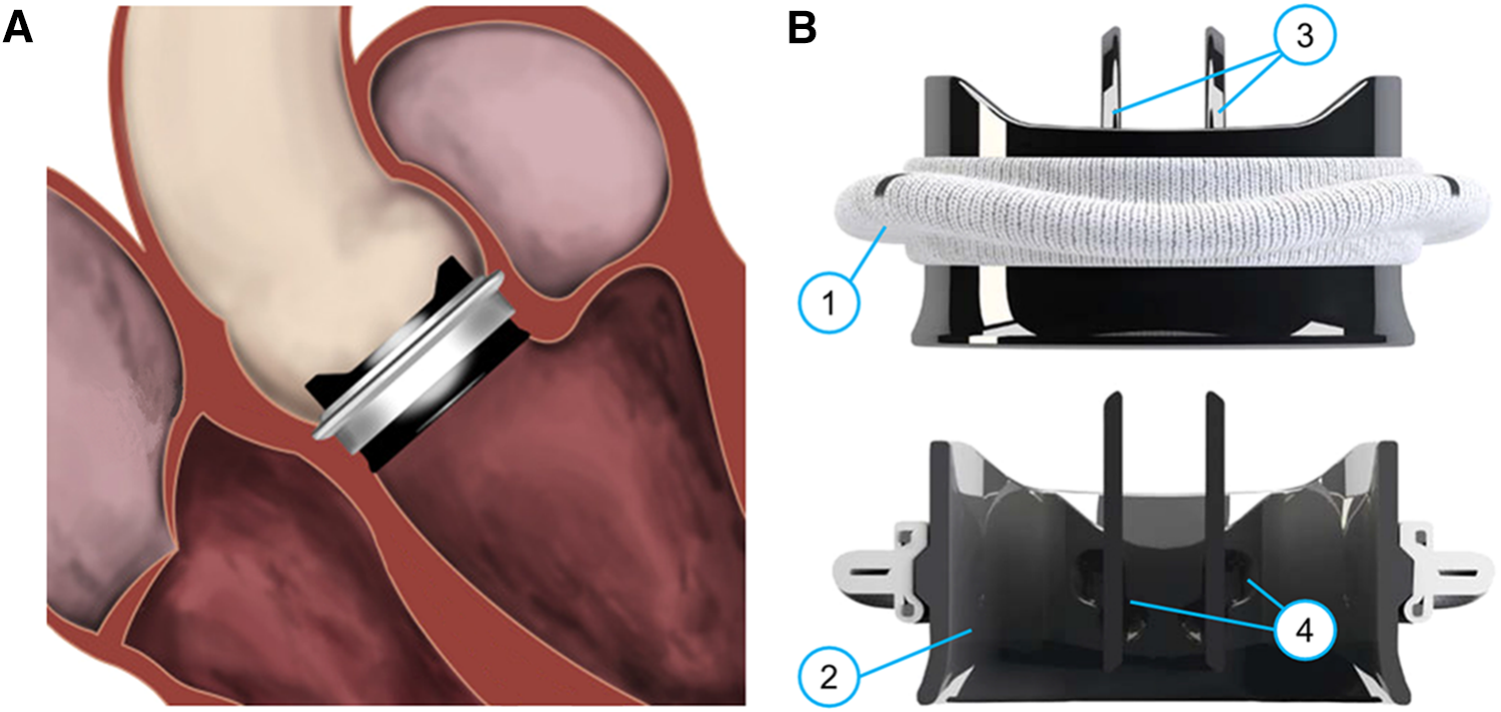
(**A)** Sketch of an implanted On-X BMAV (Artivion, Kennesaw, GA, USA), (**B**) three-dimensional and cross-section view of the same valve model. The different components of a BMAV are indicated: (1) sewing cuff, (2) valve housing, (3) leaflets, (4) hinges connecting leaflets to the housing. Panel (**A**) is adapted with permission from Dalén et al. (107), illustration by Magnus Dalén, copyright Magnus Dalén (https://creativecommons.org/licenses/by/4.0/), panel (**B**) is reprinted with permission from Jawitz et al. (108) (https://creativecommons.org/licenses/by-nc-nd/4.0/).

CM&S has been adopted to investigate the fluid dynamic behavior of various cardiovascular devices, including ventricular assist devices [e.g., (13, 14)], prosthetic heart valves [e.g., (15, 16)], vascular stents [e.g., (17–19)], intravascular catheters [e.g., (20, 21)] and stent grafts [e.g., (22, 23)]. This study specifically centers on prosthetic aortic valves, implantable devices designed to replace a native diseased aortic valve (Figure 1A). Aortic valve replacement becomes necessary due to conditions such as calcific aortic valve disease, aortic stenosis, and congenital aortic valve diseases (24, 25). Aortic valve prostheses emulate the functionality of the native valve, with leaflets that open and close during the cardiac cycle based on the transvalvular pressure drop. This mechanism regulates the flow of blood from the left ventricle into the aorta, minimizing retrograde flow.

Prosthetic valve leaflets can be composed of either biological tissues or inorganic materials. In the latter case, the valve prosthesis is referred to as mechanical aortic valve. Mechanical aortic valves are known for their high durability and low re-operation rates (26), making them suitable for aortic valve replacement in relatively young patients. Specifically, according to international guidelines, they are recommended over biological valves for surgical aortic valve replacement in patients younger than 60 years old (25). Current mechanical aortic valves feature a bileaflet design (Figure 1B). A bileaflet mechanical aortic valve (BMAV) comprises a sewing cuff for surgical attachment near the aortic annulus (Figure 1B, component 1), a rigid housing determining the shape of valve orifice (Figure 1B, component 2), and two rigid leaflets (Figure 1B, components 3). Each leaflet is connected to the BMAV housing through two hinges (Figure 1B, components 4) and rotates around the axis defined by the hinges in response to the transvalvular pressure drop.

BMAVs design markedly impacts on fluid dynamics, generating flow patterns that substantially differ from physiological ones (27, 28). Such altered fluid dynamics increases the risk of platelet activation and damage to blood cells, potentially leading to thromboembolic events and requiring the use of anticoagulation therapy (27, 28). According to the standard ISO 5840:2021 (29, 30), BMAV fluid dynamics should be accurately investigated to demonstrate adequate valve performance in terms of effective orifice area (EOA) and regurgitant fraction (RF), and to ensure low thrombogenicity. Fluid dynamic studies of BMAVs are typically conducted experimentally. In particular, the flow through BMAVs is replicated *in vitro* with pulse duplicators and it can be visualized and quantified using laser velocimetry techniques, with particle image velocimetry (PIV) considered the experimental gold standard. However, PIV-based approaches are extremely time-consuming and require an expensive equipment, in particular when four-dimensional (4D) velocity measurements have to be performed to fully characterize the fluid dynamics through BMAVs. Therefore, it is advisable to complement PIV measurements with CM&S, which can provide 4D flow fields with adequate spatial and temporal resolution, enabling comprehensive fluid dynamic analyses and thromboembolic risk prediction (31, 32). The integration of experimental and computational approaches is particularly recommended for evaluating thrombogenicity and hemolysis risk associated with heart valve prostheses (29).

BMAV opening and closure is dictated by the fluid dynamic forces experienced by valve leaflets and, in turn, leaflets shape and position influence blood flow through the valve. Therefore, simulating this interaction between BMAVs and blood is crucial for a comprehensive *in silico* study of the kinematics and fluid dynamics of these devices. The fluid-structure interaction (FSI) computational approach is based on simulating the dynamics of mechanical systems characterized by the interaction between structural and fluid components. In FSI studies, both the structural mechanics and the fluid dynamics are solved, and information is exchanged between the two domains (32). Thanks to these features, FSI simulation has emerged as the most comprehensive approach for investigating BMAVs *in silico* (33).

In this context, the aims of the present narrative review are twofold. The first aim is to report the state of the art of FSI simulations of BMAVs. The variety of FSI modelling strategies adopted in the literature is described from the methodological viewpoint, with particular focus on the applications of BMAVs simulations. The second aim is to describe how FSI simulations can be implemented in total product life cycle of BMAVs, specifically how they can be used to complement and partially replace *in vitro* testing in the preclinical and post-market phases. Since simulation credibility is essential to make the use of CM&S viable in regulatory pathways, this section also presents how FSI simulations of BMAVs can be conducted according to the transparency and consistency requirements proposed by FDA and ASME guidelines, particularly in terms of CM&S verification, validation, and uncertainty quantification (VVUQ) (11, 12).

## 2 State of the art of FSI simulation of mechanical aortic valves

### 2.1 Modelling approaches

The general workflow of FSI analysis follows the typical steps of computational modelling, namely creation of a geometrical model, discretization of the geometrical model, analysis set-up, simulation, and post-processing of the results (3). The creation of the geometrical model and the analysis set-up require introducing modelling strategies to properly represent the physical reality to be reproduced. In FSI simulations of BMAVs, modelling strategies mainly involve (i) geometry definition, (ii) blood rheological model and blood flow regime, with the potential introduction of turbulence models, (iii) boundary conditions and initial conditions, and (iv) the FSI numerical schemes. In the following sections, the modelling choices adopted in the literature to couple with these aspects are described. The modelling approaches adopted in each FSI study available on BMAVs are also summarized in Tables 1, 2. Specifically, Table 1 describes the approaches concerning geometry definition, blood rheological model, flow regime, and boundary conditions, while Table 2 reports the FSI methodologies and the utilized solvers.

**Table 1:** Modelling strategies in FSI simulations of BMAVs: geometry definition, rheology, turbulence modelling, and boundary conditions.

First Author, year (reference)	3D/2D	Ventricle model	BMAV housing	BMAV design	Wall deformability	Geometry downstream of BMAV	Rheology	Turbulence model	Inlet BC	Outlet BC
Nowak, 2023 (34)	3D	No	No	NA	NA	Patient-specific aorta, idealized aortic root	Carreau	Laminar vs. k-ω SST vs. realizable k-ε	Velocity	Windkessel vs. 0 Pa
Nitti, 2022 (31)	3D	No	Yes	St Jude Medical Regent	No	Straight with sinuses	Newtonian	DNS	Flow rate	Non reflecting
Gallo, 2022 (27)	3D	No	No	St Jude Regent	No	Straight with sinuses	Newtonian	DNS	Flow rate	Non reflecting
Asadi, 2022 (35)	3D	Yes	Yes	St Jude Regent	No	Patient-specific aorta	Newtonian	NA	Ventricle model	Convective, velocity
Ahmed, 2022 (36)	2D	No	No	NA	NA	Straight with sinuses	Generalized cross model	SST k-w	Pressure waveform	Pressure waveform
Kolahdouz, 2021 (37)	3D	No	Yes	St Jude Medical Regent	NA	Straight with sinuses	Newtonian	NA	Windkessel, normal traction/zero tangential velocity	Windkessel, normal traction/zero tangential velocity
Sadipour, 2020 (38)	3D	No	Yes	On-X	Yes vs. no	Patient-specific	Newtonian	Realizable k-ε with enhanced wall function	Flow rate	Pressure waveform
Abbas, 2020 (39)	3D	No	Yes	Medtronic ATS	NA	Straight with and without sinuses	Carreau-Yasuda	Laminar	Velocity	NA
Abbas, 2020 (40)	3D	No	Yes	NA	NA	Straight with and without sinuses	Carreau-Yasuda	Laminar	Velocity	Pressure waveform
Yeh, 2019 (41)	3D	No	Yes	NA (commercially available)	No	Straight with sinuses	Quemada	Laminar	Pressure waveform	Pressure waveform
Spühler, 2018 (42)	3D	Yes	No	Idealized	NA	Straight	Newtonian	NA	Ventricle model	NA
Banks, 2018 (43)	2D, 3D	No	No	NA	NA	Straight	Newtonian	NA	Pressure waveform, no transverse velocity	Zero pressure
Zhou, 2016 (44)	3D	No	Yes	NA	NA	Straight with bulge	Newtonian	Laminar, k-ε	Velocity (steady vs. waveform)	Zero pressure
Mirkhani, 2016 (45)	3D	No	Yes	On-X	No	Patient-specific	Newtonian	Realizable k-ε	Flow rate	Pressure waveform
De Vita, 2016 (46)	3D	No	Yes	Sorin	NA	Straight with sinuses	Newtonian vs. Carreau-Yasuda	DNS	Flow rate	NA
Annerel, 2015 (47)	3D	Yes	Yes	Carbomedics standard heart valve	No	Patient-specific vs. straight	Newtonian	NA	Ventricle model	Pressure waveform at descending aorta, flow rate at aortic arch side branches
Annerel, 2014 (48)	3D	No	Yes	Carbomedics standard heart valve	NA	Straight with sinuses	Newtonian	Implicit LES	Flow rate	Constant pressure
Borazjani, 2013 (49)	3D	Yes	Yes	Regent	No	Patient-specific	NA	NA	Ventricle model	NA
Le, 2013 (50)	3D	Yes	Yes	Regent	No	Patient-specific	Newtonian	NA	Ventricle model	NA
Li, 2012 (51)	3D	No	Yes	St Jude Medical	NA	Straight with bulge	Newtonian	k-ω	Velocity	Pressure waveform
Kim, 2012 (52)	3D	No	Yes	St Jude standard	NA	Straight with sinuses	Newtonian	Standard k-ε	Flow rate	Zero pressure
De Tullio, 2012 (53)	3D	No	Yes	St Jude Medical	Yes	Straight vs. straight with bulge	Newtonian	DNS	Pressure and flow rate	NA
Annerel, 2012 (54)	3D	Yes, no	Yes	ATS open pivot standard heart valve	No	Straight	Newtonian	Laminar	Velocity vs. contracting ventricle	Pressure
Annerel, 2012 (55)	3D	No	Yes	ATS open pivot standard heart valve	No	Straight with and without sinuses	Newtonian	NA	Flow pulse	Pressure profile
Annerel, 2012 (56)	3D	No	Yes	ATS open pivot standard heart valve	No	Straight with and without sinuses	Newtonian	Laminar	Flow pulse	Pressure profile
Hong, 2011 (57)	3D	No	Yes	St Jude bileaflet (standard) model	NA	Straight with sinuses	Newtonian	Standard k-ε	Flow rate	Zero pressure
De Tullio, 2011 (58)	3D	No	Yes	Sorin Bicarbon	No	Straight vs. straight with bulge vs. straight with sinuses	Newtonian	DNS	Velocity	Resistance
De Tullio, 2011 (59)	3D	No	Yes	Sorin Bicarbon	Yes	Straight vs. straight with bulge	Newtonian	DNS	Pressure and velocity	Coronary porosity
Simon, 2010 (60)	3D	No	Yes	St Jude Medical, Carbomedics	NA	Straight with bulge	Newtonian	Laminar	Velocity	NA
Borazjani, 2010 (61)	3D	No	Yes	St Jude Regent	NA	Patient-specific	Newtonian	NA	Flow rate	Convective
Borazjani, 2010 (62)	3D	No	Yes	St Jude Regent	No	Patient-specific	Newtonian	NA	Flow rate	Convective
Annerel, 2010 (63)	3D	No	Yes	ATS open pivot standard heart valve	No	Straight with sinuses	NA	Laminar	Flow pulse	Constant pressure
Xia, 2009 (64)	3D	No	Yes	St Jude	No	Straight	Newtonian	Laminar	Velocity	Constant pressure
Morbiducci, 2009 (28)	3D	No	Yes	St Jude Hemodynamic Plus	No	Straight with sinuses	Newtonian	DNS	Velocity	Stress-free
Hong, 2009 (65)	3D	No	Yes	St Jude Medical	NA	Straight with sinuses	Newtonian	NA	Flow rate	Zero pressure
Guivier-Curien, 2009 (66)	3D	No	Yes	St Jude	No	Straight with bulge	Newtonian	Laminar	Velocity	Free conditions
De Tullio, 2009 (67)	3D	No	Yes	Sorin Bicarbon	NA	Straight with sinuses	Newtonian	DNS	Velocity	NA
Choi, 2009 (68)	3D	No	Yes	St Jude Medical	NA	Straight with sinuses	Newtonian	Laminar	Pressure waveform	Zero pressure
Nobili, 2008 (33)	3D	No	Yes	St Jude	No	Straight with sinuses	Newtonian	DNS	Velocity	NA
Borazjani, 2008 (69)	3D	No	Yes	St Jude Regent	NA	Straight with bulge	NA	NA	Flow	Convective
Tai, 2007 (70)	3D	No	Yes	St Jude Medical	NA	Straight	NA	NA	Velocity	NA
Palmieri, 2007 (71)	3D	No	Yes	Sorin Bicarbon	NA	Straight with sinuses	Newtonian	Standard k-ε	Pressure	NA
Nobili, 2007 (72)	3D	No	Yes	St Jude Hemodynamic Plus	NA	Straight with bulge	Newtonian	Laminar	Pressure waveform	Zero pressure
Guivier, 2007 (73)	2D	No	No	St Jude Medical Hemodynamic Plus	NA	Straight with sinuses	Newtonian	Laminar	Velocity	Zero pressure
Ge, 2007 (74)	3D	No	No	ST Jude Regent	NA	Straight with sinuses	NA	NA	Velocity	NA
Dumont, 2007 (75)	3D	No	Yes	ATS open pivot, St Jude Medical Regent	NA	Straight	Newtonian	Laminar	Velocity	NA
Dasi, 2007 (76)	3D	No	Yes	St Jude Medical Regent	No	Straight with bulge	NA	DNS	Flow rate	NA
Yang, 2006 (77)	3D	No	Yes	St Jude Medical Standard	No	Straight with bulge	NA	LES	Flow rate	Convective
Bang, 2006 (78)	3D	No	Yes	Edward TEKNA	NA	Straight with and without sinuses	Newtonian	NA	Pressure waveform	Pressure waveform
Dumont, 2005 (79)	3D	No	Yes	AP ATS open pivot	NA	Straight	NA	NA	Flow rate	NA
Bang, 2005 (80)	3D	No	Yes	Edward TEKNA	Yes	Straight with sinuses	Newtonian	NA	Pressure waveform	Pressure waveform
Redaelli, 2004 (81)	3D	No	Yes	St Jude Medical Hemodynamic Plus	No	Straight with bulge	Newtonian	Laminar	Pressure waveform	Zero pressure
Shi, 2003 (82)	3D	No	NA	St Jude Medical	NA	Straight	Newtonian	Laminar	Velocity	Constant pressure, zero gradients of other variables
Choi, 2003 (83)	3D	No	Yes	St Jude Medical	NA	Straight with sinuses	Newtonian	Laminar	Pressure waveform	Pressure waveform
Choi, 2001 (84)	2D	No	Yes	St Jude Medical	NA	Straight with sinuses	Newtonian vs. Carreau	Laminar	Pressure waveform	Pressure waveform
Choi, 2001 (85)	2D	No	Yes	St Jude Medical	NA	Straight with sinuses	Newtonian vs. Carreau	Laminar	Pressure waveform	Pressure waveform

BC, boundary condition; BMAV, bileaflet mechanical aortic valve; DNS, direct numerical simulation; LES, large-eddy simulation; NA, not accessible; SST, shear stress transport.

**Table 2:** FSI algorithms and solvers used in FSI studies of BMAVs.

First Author, year (reference)	Partitioned vs. monolithic	Coupling	Kinematic description	Flow solver	Structural solver
Nowak, 2023 (34)	Partitioned	NA	Boundary conforming	FVM (Ansys Fluent)	NA
Nitti, 2022 (31)	Partitioned	Strong	Non-boundary conforming	FDM (in-house solver)	FEM (in-house solver)
Gallo, 2022 (27)	Partitioned	Strong	Non-boundary conforming	FDM (in-house solver)	FEM (in-house solver)
Asadi, 2022 (35)	Partitioned	Strong	Non-boundary conforming	FVM (in-house solver)	In-house solver
Ahmed, 2022 (36)	Partitioned	Strong	Boundary conforming	FVM (Ansys Fluent)	NA
Kolahdouz, 2021 (37)	Partitioned	NA	Non-boundary conforming	FDM (NA)	NA
Sadipour, 2020 (38)	Partitioned	Strong	Boundary conforming	FVM (Ansys Fluent)	FEM (Ansys Mechanical)
Abbas, 2020 (39)	Partitioned	Strong	Boundary conforming	FVM (Ansys Fluent)	FVM (in-house developed subroutines)
Abbas, 2020 (40)	Partitioned	Strong	Boundary conforming	FVM (Ansys Fluent)	FVM (in-house developed subroutines)
Yeh, 2019 (41)	Partitioned	NA	Boundary conforming	FEM (COMSOL Multiphysics)	FEM (COMSOL Multiphysics)
Spühler, 2018 (42)	Monolithic	–	Boundary conforming	FEM (in-house solver)	FEM (in-house solver)
Banks, 2018 (43)	Partitioned	NA	Boundary conforming	FVM (NA)	NA
Zhou, 2016 (44)	Partitioned	Weak	Boundary conforming	FVM (Ansys Fluent)	NA
Mirkhani, 2016 (45)	Partitioned	Strong	NA	FVM (Ansys Fluent)	FEM (Ansys Mechanical)
De Vita, 2016 (46)	Partitioned	Strong	Non-boundary conforming	In-house solver	In-house solver
Annerel, 2015 (47)	Partitioned	Strong	Boundary conforming	FVM (Ansys Fluent)	FVM (in-house developed subroutines)
Annerel, 2014 (48)	Partitioned	Strong	Boundary conforming	FVM (Ansys Fluent)	FVM (in-house developed subroutines)
Borazjani, 2013 (49)	Partitioned	Strong	Non-boundary conforming	FVM (in-house solver)	In-house solver
Le, 2013 (50)	Partitioned	Strong	Non-boundary conforming	FVM (in-house solver)	In-house solver
Li, 2012 (51)	Partitioned	NA	NA	FVM (Ansys Fluent)	NA
Kim, 2012 (52)	NA	NA	Boundary conforming	FVM (Ansys Fluent)	NA
De Tullio, 2012 (53)	Partitioned	Strong (BMAV), weak (wall)	Non-boundary conforming	FDM (in-house solver)	In house solver, FEM (Ansys Multiphisics)
Annerel, 2012 (54)	Partitioned	Strong	Boundary conforming	NA	NA
Annerel, 2012 (55)	Partitioned	Strong	Boundary conforming	FVM (Ansys Fluent)	NA
Annerel, 2012 (56)	Partitioned	Strong	Boundary conforming	FVM (Ansys Fluent)	FVM (in-house developed subroutines)
Hong, 2011 (57)	Partitioned	Strong	Boundary conforming	FVM (Ansys Fluent)	NA
De Tullio, 2011 (58)	Partitioned	Strong	Non-boundary conforming	FDM (in-house solver)	In-house solver
De Tullio, 2011 (59)	Partitioned	Strong (BMAV), weak (wall)	Non-boundary conforming	FDM (in-house solver)	In-house solver, FEM (Ansys Multiphisics)
Simon, 2010 (60)	NA	NA	Non-boundary conforming	FVM (in-house solver)	In-house solver
Borazjani, 2010 (61)	Partitioned	Strong	Non-boundary conforming	FVM (in-house solver)	In-house solver
Borazjani, 2010 (62)	Partitioned	NA	Non-boundary conforming	FVM (in-house solver)	In-house solver
Annerel, 2010 (63)	Partitioned	Strong	Boundary conforming	FVM (Ansys Fluent)	FVM (in-house developed subroutines)
Xia, 2009 (64)	Partitioned	Weak	Non-boundary conforming	FVM (in-house solver)	In-house solver
Morbiducci, 2009 (28)	Partitioned	Strong	Boundary conforming	FVM (Ansys Fluent)	FVM (in-house developed subroutines)
Hong, 2009 (65)	Partitioned	Strong	Boundary conforming	FVM (Ansys Fluent)	FVM (in-house developed subroutines)
Guivier-Curien, 2009 (66)	Partitioned	Strong	Boundary conforming	FVM (Ansys Fluent)	NA
De Tullio, 2009 (67)	Partitioned	Strong	Non-boundary conforming	FDM (in-house solver)	In-house solver
Choi, 2009 (68)	Partitioned	Strong	Boundary conforming	FVM (ESI Group CFD-ACE)	FEM (ESI Group FEMSTRESS)
Nobili, 2008 (33)	Partitioned	Strong	Boundary conforming	FVM (Ansys Fluent)	FVM (in-house developed subroutines)
Borazjani, 2008 (69)	Partitioned	Weak vs. strong	Non-boundary conforming	FVM (in-house solver)	In-house solver
Tai, 2007 (70)	Partitioned	Weak	Non-boundary conforming	FVM (in-house solver)	In-house solver
Palmieri, 2007 (71)	Partitioned	Strong	NA	FVM (Ansys Fluent)	FVM (in-house developed subroutines)
Nobili, 2007 (72)	Partitioned	Weak vs. strong	Boundary conforming	FVM (Ansys Fluent)	FVM (in-house developed subroutines)
Guivier, 2007 (73)	Partitioned	Strong	Boundary conforming	FVM (Ansys Fluent)	FVM (in-house developed subroutines)
Ge, 2007 (74)	NA	NA	Non-boundary conforming	FVM (in-house solver)	In-house solver
Dumont, 2007 (75)	Partitioned	Strong	NA	FVM (Ansys Fluent)	FVM (in-house developed subroutines)
Dasi, 2007 (76)	NA	NA	Non-boundary conforming	In-house solver	In-house solver
Yang, 2006 (77)	NA	NA	Non-boundary conforming	FDM (in-house solver)	NA
Bang, 2006 (78)	Partitioned	NA	NA	FVM (ESI Group CFD-ACE+)	FEM (ESI Group FEMSTRESS)
Dumont, 2005 (79)	Partitioned	Strong	Boundary conforming	FVM (Ansys Fluent)	FVM (in-house developed subroutines)
Bang, 2005 (80)	Partitioned	NA	NA	FVM (ESI Group CFD-ACE+)	FEM (ESI Group FEMSTRESS)
Redaelli, 2004 (81)	Partitioned	Weak	Boundary conforming	FVM (Ansys Fluent)	FVM (in-house developed subroutines)
Shi, 2003 (82)	NA	NA	NA	In-house solver	In-house solver
Choi, 2003 (83)	Partitioned	NA	NA	FVM (ESI Group CFD-ACE+)	FEM (ESI Group FEMSTRESS)
Choi, 2001 (84)	Partitioned	Strong	NA	FVM (ESI Group CFD-ACE+)	FEM (ESI Group FEMSTRESS)
Choi, 2001 (85)	Partitioned	Strong	NA	FVM (ESI Group CFD-ACE+)	FEM (ESI Group FEMSTRESS)

FDM, finite difference method; FEM, finite element method; FVM, finite volume method; NA, not accessible.

#### 2.1.1 Geometry definition

**Figure 2:**
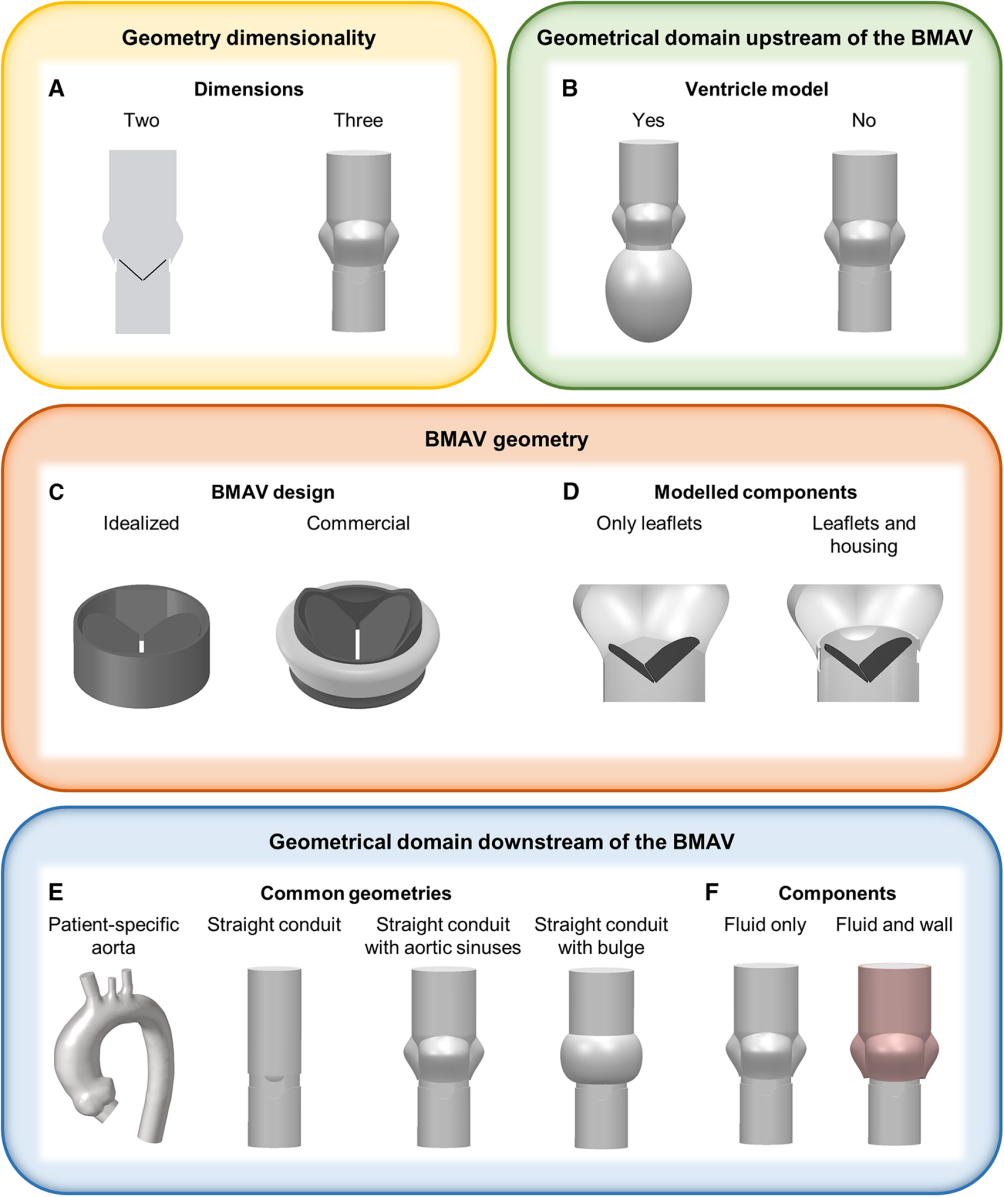
Main geometrical modelling strategies for FSI simulations of BMAVs, concerning (**A**) geometry dimensionality, (**B**) ventricle geometry, (**C**) BMAV design, (**D**) modelled BMAV components, (**E**) common geometrical models downstream of the BMAV and (**F**) components downstream of the BMAV.

The most relevant aspects in defining the geometrical model for FSI simulations of BMAVs include the geometry of the valve itself, and the geometry of the fluid and solid domain upstream and downstream of the valve (Figure 2). A crucial consideration involves deciding whether to represent the entire geometry under investigation as a two-dimensional (2D) or three-dimensional (3D) geometrical model (Figure 2A). Since the flow structures downstream of a BMAV are highly 3D (33, 86), the vast majority of FSI studies opted for a 3D representation (27, 28, 31, 33–35, 37–72, 74–83) (Table 1). Nevertheless, 2D simulations were conducted in previous studies focusing on the FSI approach (43, 85).

Concerning the geometrical modelling of the BMAV, in the majority of FSI studies the device’s geometry was reconstructed to replicate commercially available designs, such as the Bicarbon valve (Corcym Srl, Milan, IT) (58, 59, 67, 71), the ATS Open Pivot valve (Medtronic, Dublin, IR) (54–56, 63, 75, 79), the On-X valve (Artivion, Kennesaw, GA, USA) (38, 45) and valves from St. Jude series (Abbott Laboratories, Chicago, IL, USA) (27, 28, 31, 33, 35, 37, 49–53, 57, 60–62, 64–66, 68–70, 72–77, 81–85). Nevertheless, idealized valve models, which do not refer to specific commercial designs, are sometimes considered (Figure 2C) (42). Generally, the BMAV sewing cuff is not included in the structural domain, nor it is accounted for in determining model geometry, as it does not alter blood flow patterns (45). Consequently, the BMAV geometrical model can include valve leaflets and housing (28, 31, 33, 35, 37–41, 44–72, 75–81, 83–85) or valve leaflets only (27, 34, 36, 42, 43, 73, 74), (Table 1). Typically, only the FSI between valve leaflets and blood is of interest, and therefore only valve leaflets are included in the structural domain of the simulation. Nevertheless, valve housing geometry can be utilized to define the fluid domain geometry near the valve site so that the fluid domain does not overlap with the region occupied by the housing (Figure 2D). Additionally, different levels of detail can be adopted to model the hinges connecting valve leaflets and the housing. The hinge recess of the housing can be reproduced with a realistic geometry, featuring a characteristic butterfly shape (28, 33, 37, 38, 45, 47, 48, 60, 75). Alternatively, the coupling between the leaflets and the housing can be simplified, for example, by replacing the hinge with a peripheral gap (27, 46, 58, 67) or modelling a simplified pin (41) or spherical (72, 81) hinge. Simplified hinge geometry typically results in leaflet rotation being limited by angular constraints rather than by the blocking mechanism of the hinge. Additionally, the friction in the hinge is usually neglected (33, 35, 38, 41, 49, 55, 56, 58, 61–63, 67, 69), since it is much smaller than fluid forces and reliable coefficients are difficult to retrieve (49, 67).

The BMAV can be simply located within a straight conduit (39, 40, 42, 43, 53–56, 58, 59, 64, 70, 75, 78, 79, 82), although more realistic geometries have been explored in the literature (Table 1). In detail, concerning the upstream domain of the valve some studies considered geometrical models incorporating the left ventricle, either with patient-specific (35, 47, 49, 50) or idealized (54) geometry (Figure 2B). As for the downstream domain of the valve, both idealized and patient-specific geometrical modelling strategies have been explored (Table 1). Idealized geometries usually neglect the curvature of the ascending aorta, representing the region of interest as a straight tube. Straight tube geometries may include idealized sinuses (27, 28, 31, 33, 36, 37, 39–41, 46, 48, 52, 55–58, 63, 65, 67, 68, 71, 73, 74, 78, 80, 83–85) or surfaces of revolution (44, 51, 53, 58–60, 66, 69, 72, 76, 77, 81) in their proximal part to account for the presence of the sinuses of Valsalva (Figure 2E, Table 1), as done for *in vitro* set-ups (87, 88). Straight geometries with or without aortic sinuses may also be designed to replicate experimental set-ups (28, 33, 37, 41, 48, 66, 67, 71, 72, 76, 81) or aortic prostheses designs (53, 58, 59) (Table 1). Patient-specific geometries, usually reconstructed from magnetic resonance (34, 35, 47, 49, 50, 61, 62) or computed tomography (38, 45) images, consider the actual curvature and tortuosity of the ascending aorta, ensuring a higher level of similarity between simulated blood flow and *in vivo* conditions downstream of the valve. In the majority of idealized and patient-specific geometrical models, no structural component was included in the model downstream of the valve (Table 1), except in some studies where deformable aortic walls (38, 80) or deformable aortic valved prostheses (53, 59) were incorporated (Figure 2F). Simplifying the model by neglecting the distensibility of the aorta introduces a simplification with respect to real compliance of the vessel. However, such a simplification allows for markedly reducing the complexity and computational cost of FSI simulations, as well as for replicating *in vitro* set-ups, which usually feature rigid aortic roots.

#### 2.1.2 Rheological models and turbulence models

In physiologic conditions, blood flow in the ascending aorta might become transitional only for a brief time interval immediately after peak systole, when Reynolds number reaches values up to 4,000–5,000 (34). When a BMAV is implanted, valve leaflets and housing determine a reduction in the orifice area available for the streaming blood with respect to the physiologic case, with increased Reynolds number to confirm the development of turbulence in blood flow (34). Most FSI studies of BMAVs assumed a laminar flow regime (34, 39–41, 44, 54, 56, 60, 63, 64, 66, 68, 72, 73, 75, 81–85) (Table 1), justifying this assumption with the short duration of the time interval characterized by instantaneous Reynolds number exceeding those for laminar flow. However, the transitional or turbulent flow occurring in the flow deceleration phase immediately after peak systole motivated some authors to account for turbulence in their model, as summarized in Table 1. Currently, the direct numerical simulation (DNS) approach represents the gold standard for investigating turbulent flows through BMAVs (27, 28, 31, 33, 46, 53, 58, 59, 67, 76). DNS is a simulation approach relying on fine space and time discretization that allows for the direct resolution of the flow field at the scale where turbulent kinetic energy dissipation occurs (31). However, this approach significantly increases the computational costs of the FSI simulation. Consequently, it has mainly been adopted when the primary aim of the study involves exclusively the investigation of turbulent flow features. Large-eddy simulation (LES) or the use of turbulence models based on the Reynolds-averaged Navier-Stokes (RANS) approach represent alternatives to DNS. LES is based on directly resolving larger flow scales and modelling the smaller ones (77) and it has been adopted for simulating BMAVs both in its classical formulation (77) and with an implicit LES approach (48). Approaches based upon the RANS, where turbulence models such as κ-ε (34, 38, 44, 45, 52, 57, 71) or κ-ω models (34, 36, 51) are implemented as problem closure, have been also largely adopted for describing BMAVs fluid dynamics (Table 1) even if with debatable results, because of the inaccuracy of such models to represent low turbulence flows.

The occurrence of turbulence in the aorta in consequence of BMAV implantation is also connected to the choice of blood rheological model. As reported in Table 1, a Newtonian behavior has been commonly assumed in FSI studies of BMAVs (27, 28, 31, 33, 35, 37, 38, 42–48, 50–62, 64–68, 71–73, 75, 78, 80–85). This assumption is considered valid for blood when the flow is laminar, shear rates values are high, and the conduit through which the blood flows has a large diameter (89). The physiological shear rates and the diameter of the aortic root generally support the validity of the Newtonian assumption in FSI simulations of BMAVs (46, 89). However, the Newtonian assumption is also based on the hypothesis of flow laminarity, which is mined by the transitional or turbulent flow regimes encountered in the aortic root during systole (46). While the choice between Newtonian or non-Newtonian models does not alter macroscopic flow features (e.g., the transvalvular pressure drop), it may reflect on other analysis such as the study of the onset and dissipation of turbulence, and/or of mechanically-induced blood trauma (quantifiable in terms of hemolysis and platelet activation), for which the use of non-Newtonian models might be more appropriate (36, 46). Various shear-thinning rheological models have been explored in the literature, including Carreau model (34, 84, 85), Carreau-Yasuda model (39, 40, 46), Quemada model (41) and the generalized cross model (36). In this context, studies comparing Newtonian and non-Newtonian models demonstrated that the Newtonian assumption does not affect leaflet motions but results in an underestimation of shear stresses and mechanically-induced hemolysis (46, 84, 85).

#### 2.1.3 Boundary conditions

**Figure 3:**
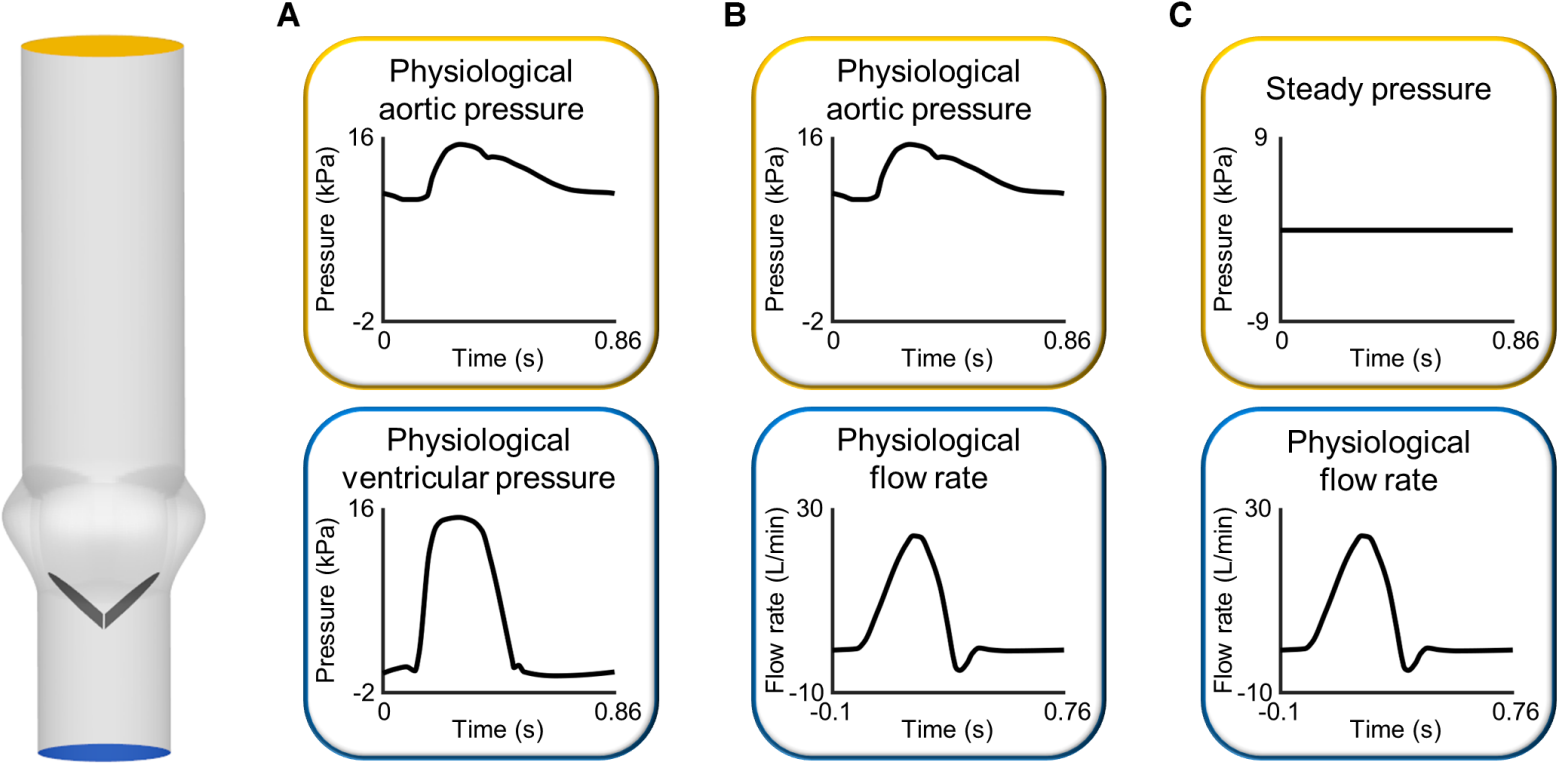
Sets of boundary conditions commonly imposed at the inlet and outlet of the fluid domain in FSI simulations of BMAVs: (**A**) pressure-pressure boundary conditions and flow rate inlet boundary conditions coupled to (**B**) unsteady and (**C**) steady pressure outlet boundary conditions. The idealized geometrical model of the aortic root has been constructed based on previous studies (109, 110).

In the vast majority of FSI studies of BMAVs (Table 1), the fluid domain typically comprises one inflow section and one outflow section, located upstream and downstream of the valve, respectively. This configuration implies a simplification of the aortic root anatomy. However, such a simplification is deemed acceptable particularly in studies involving idealized geometries and replicating experimental set-ups, which typically do not incorporate coronary arteries or aortic branches (Section 2.1.1). In a few exceptions, multiple outflow sections have been considered, incorporating coronary arteries (53, 58, 59) or aortic branches (34, 47) as part of the fluid domain. Boundary conditions are usually prescribed in terms of pressure or flow rate, as reported in Table 1. A common scenario involves prescribing pressure boundary conditions at both the inflow and outflow sections of the fluid domain (36, 41, 43, 68, 72, 78, 80, 81, 83–85). In this set-up, ventricular pressure and aortic pressure waveforms can be prescribed at inflow and outflow sections, respectively (Figure 3A). Alternatively, a transvalvular pressure drop and a steady reference pressure can be prescribed at inflow and outflow sections, respectively. In both cases, valve leaflets’ kinematics is determined by the transvalvular pressure drop, as for native aortic valves. Furthermore, pressure boundary conditions can be obtained from ventricular and aortic pressure waveforms acquired with either *in vitro* or *in vivo* measurements. Steady or unsteady pressure outlet boundary conditions can also be coupled to flow rate or velocity inlet boundary conditions (34, 38, 40, 44, 45, 48, 51, 52, 54–57, 63–65, 73, 82) (Figures 3B,C). This approach replicates how boundary conditions are typically imposed in commercially available pulse duplicators and allows for a precise control of cardiac output, which is crucial for replicating the flow conditions prescribed by international standards for BMAV hydrodynamic assessment (see Section 3.2). However, imposing an inlet flow rate boundary condition during the diastolic phase of the cardiac cycle represents a non-trivial challenge for flow solvers. Less commonly, stress-free conditions (28), non-reflecting conditions (27, 31), and Windkessel boundary conditions (34) were applied at the outflow section of the fluid domain (Table 1). Lastly, a peculiar scenario concerning inlet boundary conditions is represented by studies incorporating a left ventricle model (see Section 2.1.1) (35, 42, 47, 49, 50, 54). In these studies, blood flow upstream of the valve was dictated by the intraventricular flow resulting from mitral boundary conditions (35, 42, 47, 50) and ventricle wall movement (35, 42, 47, 49, 50, 54).

#### 2.1.4 FSI algorithms

**Figure 4:**
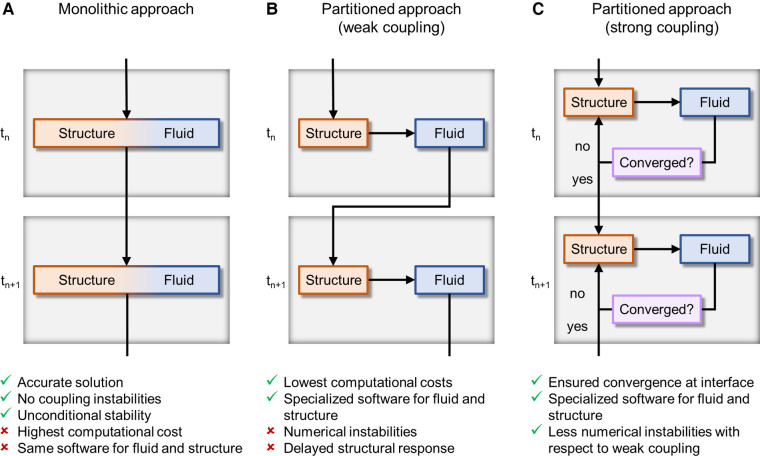
FSI algorithms adopted for simulating BMAVs, classified on the base of domains coupling approach into (**A**) monolithic algorithms and partitioned algorithms with (**B**) weak or (**C**) strong coupling. t_n_ and t_n+1_ are the current time step and the next time step, respectively.

FSI algorithms can be classified into monolithic and partitioned, according to how the structural and fluid problems are solved in relation to each other (Figure 4). Monolithic algorithms solve fluid dynamics and structural mechanics problems simultaneously by solving a single system of equations (Figure 4A) (56). Thanks to this unified treatment of the domains, there is no need to transfer information between them using a coupling strategy and unconditional stability is guaranteed (90). Unfortunately, monolithic approaches entail high computational costs and do not allow for the use of separate, specialized solvers for the fluid and structural problems (56). For this main reason, monolithic approaches have been rarely adopted to simulate BMAVs (Table 2) (42). In contrast to monolithic algorithms, partitioned FSI algorithms solve the fluid and the structural problems separately and simulate the interaction between the two domains through a coupling scheme (56, 69). This type of approach has been adopted in the vast majority of FSI simulations of BMAVs (27, 28, 31, 33–41, 43–51, 53–59, 61–73, 75, 78–81, 83–85) (Table 2). Regarding the solvers used, typically specialized solvers for either the fluid or the structural problems are adopted and integrated. Specifically, commercial flow solvers may be coupled to commercial structural solvers (38, 41, 45, 68, 78, 80, 83–85). In this regard, it is worth noting that the structural problem typically consists of a rigid rotation of the leaflets around the hinges that connect each leaflet to the housing. This aspect has enabled many authors to solve the structural problem through user defined subroutines implemented within the commercial flow solvers (Table 2). Additionally, some authors have developed in-house flow solvers for FSI simulations, mainly to respond to the need for specialized interface treatment approaches (42, 64, 67, 69, 70, 74, 77, 82) (Table 2). Partitioned algorithms couple the fluid and the structural domain either with a weak (also known as loose) or a strong coupling strategy. In the case of weak coupling, the fluid and structural problems are solved only once per time step, without checking the equilibrium of the fluid and structural solutions (Figure 4B) (56). This approach allows for reduced computational times and complexity, but it comes at the cost of a delay in the structural response to the fluid and instability rising from the added mass effect in the case of similar fluid and structural densities (90). Despite these limitations, weak coupling has been successfully adopted to simulate BMAVs (44, 64, 69, 70, 72, 81), thanks to an *ad hoc* tuning of the numerical settings (72) or using weakly coupled algorithms to simulate phases of the cardiac cycles where adequate stability is maintained (69). In contrast to weak coupling, strong coupling has been often adopted in this field (27, 28, 31, 33, 35, 36, 38–40, 45–50, 53–59, 61, 63, 65–69, 71–73, 75, 79, 84, 85) (Table 2) since it guarantees the convergence of fluid and structural results (32) and mitigates the added mass effect rising from the similarity in leaflets and blood densities. Indeed, in strong coupling the fluid and structural problems are iteratively solved within each time step until equilibrium is reached between the two domains and the convergence error falls below a certain threshold (Figure 4C) (32, 42). Although the iteration loop increases the computational cost of strong coupling compared to weak coupling, strong coupling may be preferred over weak coupling for the simulation of BMAVs because it ensures result convergence and eliminates any delay in the structural response to fluid flow (72).

**Figure 5:**
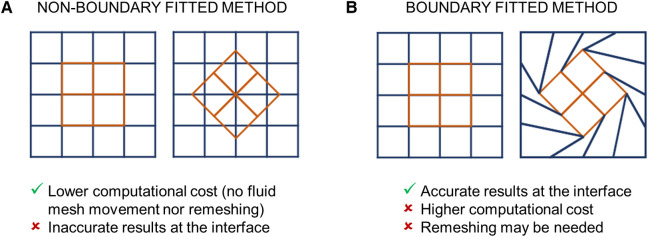
FSI algorithms adopted for simulating BMAVs, classified on the base of the kinematic description of the domains into (**A**) non-boundary fitted and (**B**) boundary fitted methods.

FSI algorithms can also be classified according to the kinematic description of the domains (56) (Figure 5). Typically, in FSI problems the structural and fluid domains are described using a Lagrangian and Eulerian approach, respectively (56). These approaches can be related through either (i) a fixed mesh approach (Figure 5A) or (ii) a moving mesh approach (Figure 5B) (32, 56). In fixed mesh approaches (also known as non-boundary fitted or non-boundary conforming methods), the fluid and structural mesh overlap and are independently defined (32, 56). The fluid mesh remains fixed in time, while the structural mesh moves according to the Lagrangian material movement. Generally, fixed mesh approaches have lower computational costs with respect to moving mesh methods, as they do not require remeshing of the fluid domain (56). However, in fixed mesh approaches the fluid mesh is non-boundary conforming, with data interpolation resulting in inaccurate interface results (32, 56). These approaches have been often used in simulating BMAVs (27, 31, 35, 37, 46, 49, 50, 53, 58–62, 64, 67, 69, 70, 74, 76, 77) (Table 2) due to high-displacement resulting from leaflet rotation, which makes fluid remeshing computationally heavy. Fixed grid approaches reported in the literature (Table 2) are mainly immersed boundary methods, which can be classified into diffused interface methods and sharp interface methods, depending on whether immersed boundaries are smeared over several nodes or not (90). Sharp interface methods applied to the simulation of BMAVs (90) include the curvilinear immersed boundary method (35, 61, 62, 69), characterized by the presence of a curvilinear background mesh, immersed interface methods (37), which specifically aim to deal with non-smooth solutions due to singular forces at the interface, and the immersed membrane method (64), enforcing velocity continuity at the interface thanks to ghost nodes (Table 2). Specifically, the investigation of fixed mesh methods for BMAV simulation aimed at improving results accuracy in the proximity of leaflet surface and reducing the numerical instabilities that affect the classical immersed boundary method, due to leaflet rigidity. In contrast to fixed mesh methods, the moving mesh approaches (also known as boundary fitted or boundary conforming methods) enable accurate interface results. Moving mesh methods are typically implemented with an arbitrary Lagrangian-Eulerian (ALE) formulation and are characterized by a moving fluid mesh, which does not overlap with the structural one, and a boundary conforming computational grid (32, 56). The movement of the fluid mesh is driven by the movement of the structure and in high-displacement FSI scenarios remeshing of the fluid domain is required to prevent mesh quality deterioration (32, 56). Moving grid methods have been widely adopted in simulating BMAVs (28, 33, 34, 36, 38–44, 47, 48, 52, 54–57, 63, 65, 66, 68, 72, 73, 79, 81) because they ensure accurate results at the fluid-solid interface, enabling for instance the analysis of wall shear stress patterns along the valve leaflets (Table 2). Alternative FSI methods, as opposed to those described above, rely on a meshless approach, approximating the solution over particles distributed throughout the domain (91). Meshless FSI approaches are currently gaining popularity (92–95) mainly because the dynamic mesh generation is not required, i.e., overcoming issues related to large deformation of the mesh. However, this poses numerical and modelling challenges (related e.g., to the connection between particles and to contact modelling) (91), which differ from those encountered in mesh-based approaches and make the adoption of meshless approaches for heart valve simulation promising but still limited (91). Therefore, studies adopting meshless FSI approaches are excluded from the present analysis.

### 2.2 Application-oriented FSI studies of BMAVs

#### 2.2.1 Blood damage prediction

One of the primary concerns associated with the use of BMAVs is the risk of mechanically-induced blood cells damage resulting from implantation (28). The altered fluid dynamics caused by BMAVs can lead to platelet activation, resulting in clot formation, and damage to red blood cells, leading to hemolysis (28). Both phenomena are linked to the magnitude of shear stress values experienced by blood cells and to the duration of exposure to them (28, 96, 97). The shear stress experienced by blood cells varies significantly within the cardiac cycle, depending on the instantaneous blood flow rate and the kinematics of BMAV leaflets. In this regard, FSI simulations account for both aspects, enabling a comprehensive assessment of the risk of mechanically induced blood damage. To this end, simulation results are typically post-processed by introducing blood damage models that quantify platelet activation state or red blood cell damage. Over the years, various blood damage models have been developed. A simple approach consists of using a model based on the accumulated product between shear stress value and time exposure, as proposed by Asadi et al. (35) and Dumont et al. (75) to evaluate platelet activation in BMAVs. Power law models for blood damage have been also developed. Specifically, Giersiepen et al. (98) proposed a power law blood damage index that can been adopted to estimate both platelet activation state and red blood cells damage by quantifying the released cytoplasm enzyme and hemoglobin, respectively (46, 60, 78). Additionally, Grigioni et al. (96) and Soares et al. (99) proposed another type of blood damage model that accounts for cells load history. These models were applied to FSI simulations of BMAVs, investigating the cumulative effect of mechanical stimuli on blood cells (28, 35, 36, 39, 67). For further details on FSI simulations of BMAVs aimed at estimating the blood clotting potential, the reader is referred to the extensive review by Zakaria et al. (97).

#### 2.2.2 Impact of BMAV design and positioning on fluid dynamics

FSI studies have been conducted to compare different valve designs, aiming to investigate the impact of BMAV geometrical features, such as hinge geometry, on blood damage risk (60, 75) or comparing the fluid dynamics of BMAVs with that of other valve prostheses, including trileaflet mechanical aortic valves (31, 51) and biological aortic valves (27). Other FSI studies have explored the impact of BMAV position in terms of rotation and/or tilt angle on fluid dynamics and valve leaflet kinematics. The relevance of BMAVs orientation in determining fluid dynamic patterns arises from the fact that BMAV design involves bilateral symmetry, whereas aortic sinuses ideally exhibit triradial symmetry, and the ascending aorta and aortic arch are non-axisymmetric (100). Different valve orientations correspond to distinct optimal fluid dynamic aspects, such as minimized turbulence (101), minimized intermittent regurgitation (61), maximized coronary flow (102) and maximized left coronary artery flow (103). Both patient-specific and idealized FSI studies have focused on valve orientation. In patient-specific aortic models, BMAV orientation was defined based on anatomical features such as the interventricular septum (47) or the aortic curvature plane (35, 61). Conversely, in idealized aortic models, BMAV orientation was defined with respect to the sinuses (40, 57, 65). Commonly considered orientations involved positioning valve leaflets symmetrically with respect to the sinuses or asymmetrically so that one leaflet directly faces one sinus. Additionally, FSI studies have also focused on BMAV title angle, which depends on the implantation procedure, pathological conditions, and tissue deformation due to tight BMAV attachment (52). The analyses have involved studying general flow features, leaflet kinematics, and risk of mechanically-induced platelet activation in the case of different tilt angles (39, 52, 65).

## 3 FSI simulations for the biomechanical evaluation of BMAVs

### 3.1 FSI simulations in BMAV total product life cycle

**Figure 6:**
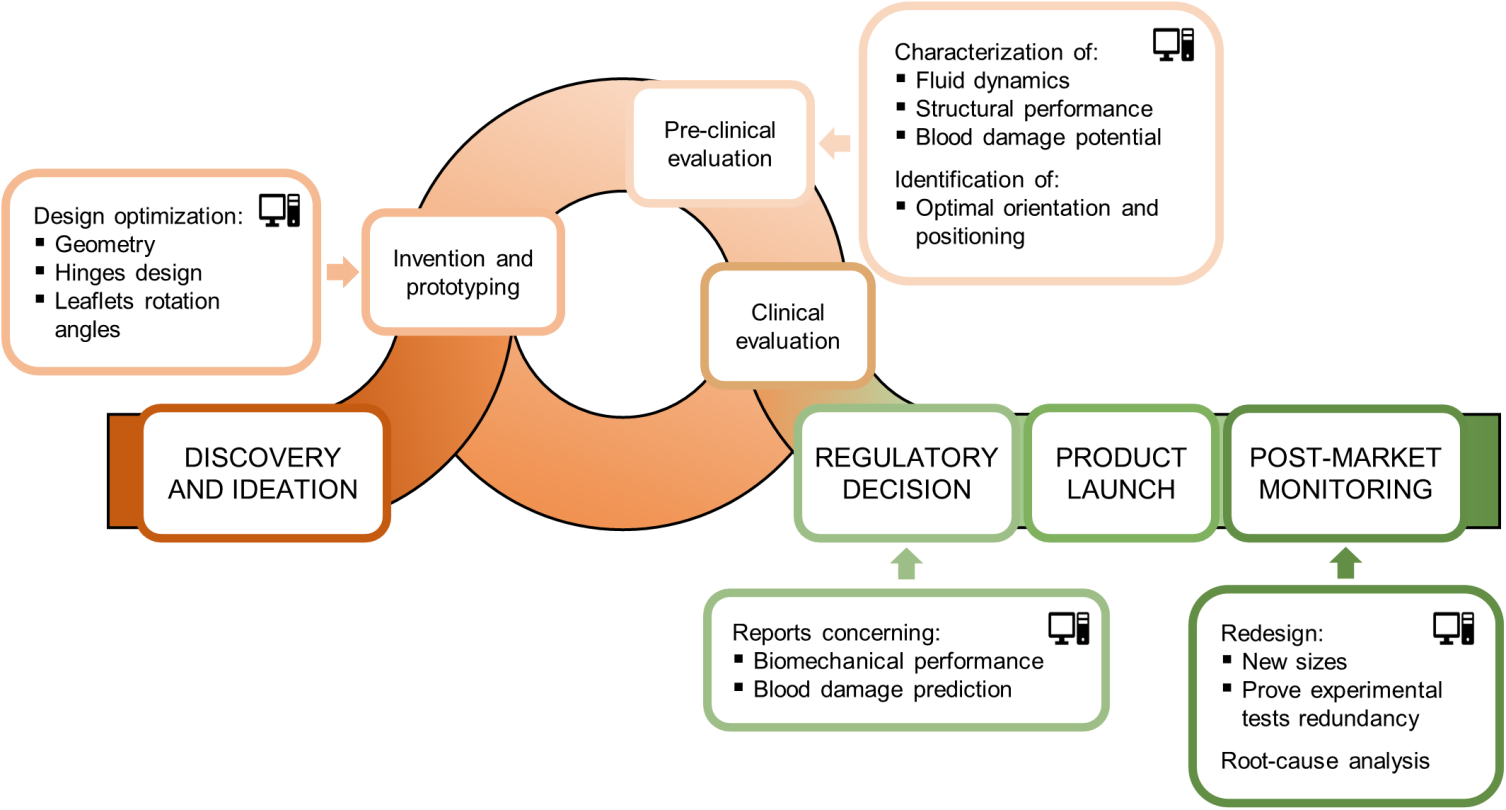
Total product life cycle of a BMAV and potential contribution of FSI simulations to its various stages.

The total product life cycle of a medical device refers to the process of device development from the ideation phase to commercialization. In this context, CM&S, and more specifically FSI simulations, can assist the various stages of BMAV total product life cycle by integrating and partially replacing experimental investigation. The most relevant stages of BMAV total product life cycle, along with the potential contribution of FSI simulations, can be listed as follows (Figure 6):

i. Device discovery and ideation: the BMAV is conceptualized, prototypes are generated, and device design is optimized. Subsequently, the BMAV is tested through preclinical and clinical investigation. During this stage, BMAVs are tested to evaluate the effect of design features on device behavior, and biomechanical aspects are investigated. Specifically, *in vitro* experiments are conducted to assess the hydrodynamic and structural performance of the device. Furthermore, other biomechanical aspects may be explored, including mechanically-induced blood damage, leaflets dynamics/kinematics and optimal valve positioning in terms implantation height with respect to the aortic annulus. At this stage, FSI simulations can be used to (at least) partially replace and/or complement *in vitro* tests, allowing for a reduction in time and cost required for device optimization.
ii. Regulatory decision: a regulatory submission is prepared and presented to a regulatory agency, incorporating results from preclinical and clinical evaluation. If FSI simulations were conducted during preclinical evaluation, simulation results predicting BMAV performance can be included in the regulatory submission to prove device safety and effectiveness.
iii. Product launch: the BMAV is placed on the market. CM&S allows to create effective visual representations of BMAVs function, aiding in scientific communication and education in academic settings, professional conferences, and educational material. Furthermore, FSI simulations have the potential to support marketing campaigns by providing engaging visual content to be used in promotional materials, presentations, and websites to attract stakeholders, including healthcare professionals, patients, and investors.
iv. Post-market monitoring: during widespread market use, the device is subject to monitoring activities to detect adverse events. FSI simulations can aid in root-cause analysis or reduce the number of experimental investigations required for design changes based on large-scale clinical implantation (1). In the case of BMAVs, post-market design changes may include the introduction of new valve sizes or modifications in BMAV sewing cuff geometry, which should result in minor changes to BMAV biomechanical performance.

Among the different stages of BMAV total product life cycle, FSI simulations can play a major role particularly in the phase of device discovery and ideation. Specifically, simulations can effectively support preclinical evaluation of the device by assessing *in silico* biomechanical aspects. Therefore, Section 3.2 elaborates on how FSI simulations can be integrated within BMAV preclinical assessment, by replicating and partially replacing *in vitro* tests. Additionally, Section 3.3 outlines the process of assessing the credibility of FSI simulations, ensuring that the results of these *in silico* tests can be utilized to support device regulatory submission.

### 3.2 FSI simulations for preclinical biomechanical evaluation of BMAVs

**Figure 7:**
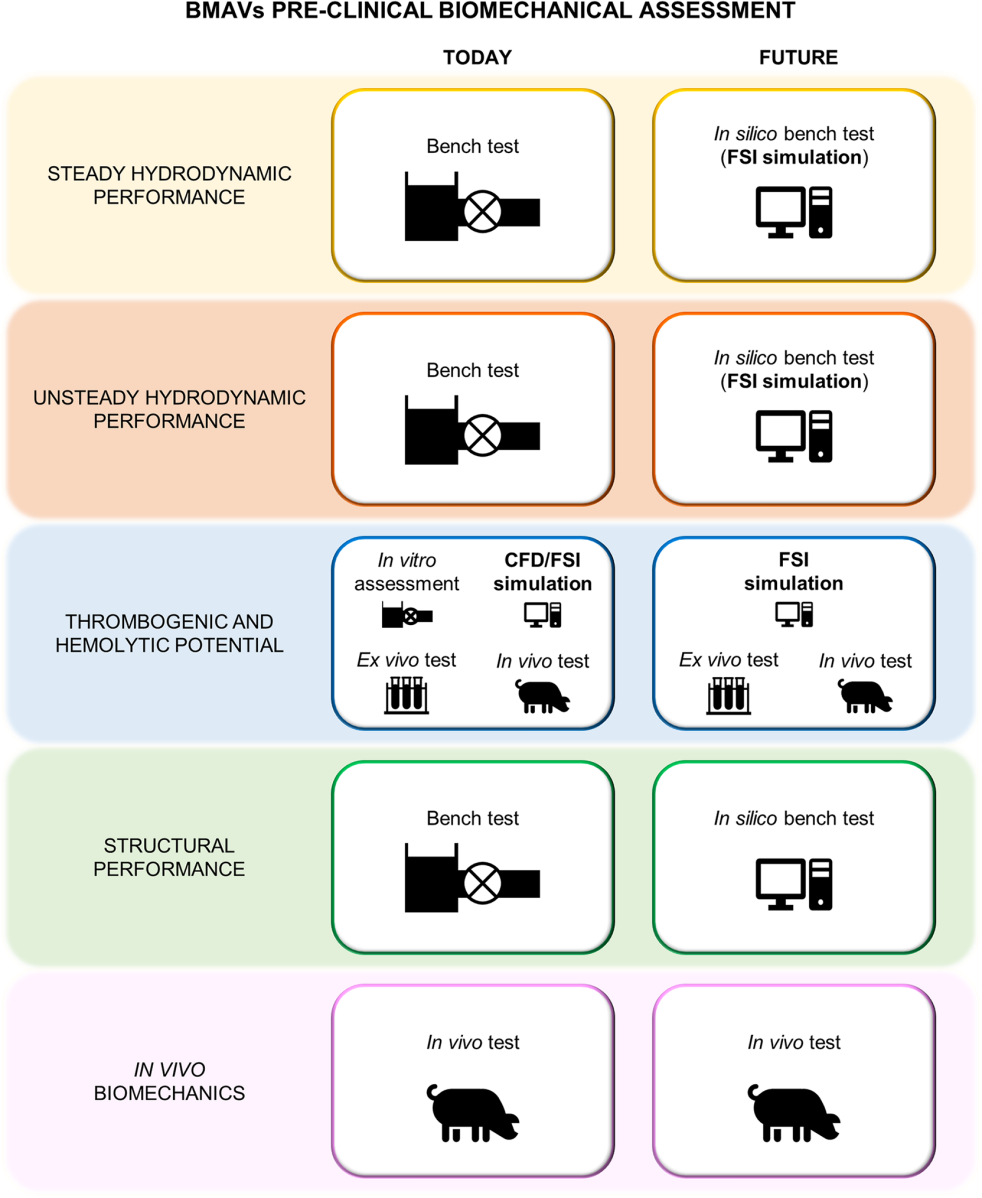
Current and future preclinical biomechanical investigation of BMAVs according to ISO 5840:2021.

Standard ISO 5840:2021 (29, 30, 104) proposes a risk-based approach to evaluate the design and manufacture of heart valve prostheses, making it a crucial guideline for the development of BMAVs. This standard describes methods for demonstrating that prosthetic heart valves meet design inputs and user needs, covering both preclinical and clinical evaluations. Specifically, the preclinical assessment includes *in vitro* and *in vivo* tests and it involves characterizing the biomechanical behavior of the valve in terms of hydrodynamic performance, structural performance and thrombogenic and hemolytic potential. As mentioned earlier, using CM&S to complement and, to some extent, replace experimental investigation has the potential to reshape the path of preclinical biomechanical evaluation of BMAVs. In this regard, Figure 7 shows current and future scenarios, emphasizing the role of CM&S in reducing *in vitro* tests. Among various CM&S approaches, FSI simulations can offer insights into the assessment of BMAV hydrodynamics (Section 2.2.2) and blood damage potential (Section 2.2.1). In this context, Table 3 summarizes the most relevant features of FSI simulations intended for replicating the experimental investigation conditions suggested by standard ISO 5840:2021, as discussed in the following paragraphs.

**Table 3:** Key features of FSI simulations replicating *in vitro* tests as defined by ISO 5840:2021 for preclinical assessment of hydrodynamic performance, thrombogenic, and hemolytic potential of surgical prosthetic heart valves.

Simulation purpose	Corresponding *in vitro* test	Fluid domain geometrical model	Boundary conditions	Main quantities of interest
Steady hydrodynamic performance assessment	Forward flow testing	Tube 35 mm inner diameter	Flow rate 5:5:30 L/min	Forward flow pressure difference, EOA
Steady hydrodynamic performance assessment	Back flow leakage testing	NA	Five equidistant back pressures	Leakage volume flow rate
Minimum hydrodynamic performance assessment	Pulsatile hydrodynamic tests	Pulse duplicator	Beat rate 70 cycles/min, cardiac output 5.0 L/min, systolic duration 35%, normotensive conditions	EOA, total RF
Pulsatile hydrodynamic performance assessment	Pulsatile hydrodynamic tests	Pulse duplicator	See (30) Annex F	Pressure difference, regurgitant volume
Thrombogenic/hemolytic potential assessment	*In vitro* flow field assessment	Pulse duplicator	Low and elevated cardiac output at 70 beats/min and 35% systolic duration, *in vivo* boundary conditions	Shear rates, platelet activation, wall shear stresses, washout time/recirculation/separation, blood damage indexes

EOA, effective orifice area; NA, not accessible; RF, regurgitant fraction.

According to ISO 5840:2021 (29, 30), BMAV hydrodynamic performance must be investigated *in vitro* under both steady and pulsatile flow conditions. Steady flow tests provide a consistent method for comparing the hydrodynamics of different BMAVs, specifically through the quantification of the steady forward flow and of the back flow leakage (29). FSI simulation could potentially substitute the *in vitro* steady-flow characterization if the experimental set-up and prescribed boundary conditions are appropriately replicated. Nevertheless, given the stationarity of these simulations, a fixed leaflets approach, and consequently a conventional computational fluid dynamics simulation with fixed walls, would likely achieve the same purpose without the need for increased simulation complexity involving FSI. Conversely, the FSI approach becomes essential when considering the replacement of pulsatile flow *in vitro* tests with *in silico* simulations. According to the ISO standard (30), pulsatile flow tests must be conducted to prove that the BMAV meets minimum hydrodynamic requirements in terms of EOA and RF. In particular, the EOA is defined as the orifice area derived from flow and pressure or velocity data, while the RF is the regurgitant volume expressed as a percentage of the forward flow volume (29). Minimum performance requirements define the minimum acceptable EOA and the maximum acceptable total RF values for a heart valve prosthesis based on its size and on whether the prosthesis is intended for mitral or aortic replacement. Standard ISO 5840:2021 defines minimum performance requirements for tests conducted at a beat rate of 70 cycles/min, a cardiac output of 5.0 L/min, 35% systolic duration and normotensive pressure conditions. Additionally, pulsatile tests should be conducted also under different conditions to characterize the device in terms of regurgitant volume and pressure difference (30). These tests must be conducted using a pulse duplicator with properly characterized performance. Therefore, the ISO standard provides some reference values for pulse duplicator performance characterization, derived from a study using St. Jude Masters Series mechanical valves (30). FSI simulations can replace or complement *in vitro* tests for pulsatile hydrodynamic assessment, provided that the geometrical domain accurately reproduces commercial pulse duplicators (see Section 2.1.1). Moreover, ensuring appropriate boundary conditions enables simulations to be conducted under the same conditions of experimental tests. Lastly, the reliability of simulations results can be tested by conducting simulations on a St. Jude Masters valve to determine whether *in silico* tests meet the performance criteria recommended for pulse duplicators.

Concerning the thrombogenic and hemolytic potential of a heart valve prosthesis, the ISO standard suggests to use an integrated approach considering both experimental and computational methods. As an example, the standard proposes a framework integrating experimental flow field assessment conducted via PIV, with CM&S, *ex vivo* flow testing, and preclinical *in vivo* testing. In this framework, the computational flow field assessment should reproduce as closely as possible the *in vivo* or *in vitro* set-up and it should specifically identify locations particularly susceptible to blood damage as well as valve features increasing the risk for thromboembolic events and hemolysis. Specifically, simulation results can be analyzed in terms of various quantities related to blood damage, including shear rates and wall shear stress (see Section 2.2.1). Given the complexity of the processes leading to thrombogenesis and hemolysis, it is unlikely that *in silico* methods can provide a comprehensive assessment without being integrated with other approaches. Nevertheless, in the framework proposed in the ISO standard, the experimental flow fields investigation is mainly proposed to visualize flow fields and validate computational simulations results. Therefore, using a previously validated simulation framework would allow for at least partially omitting *in vitro* tests. Simulation results should still be integrated with *ex vivo* and *in vivo* tests to consider the chemical and biological interaction of the BMAV with blood and surrounding biological environment.

### 3.3 Credibility assessment of FSI simulations

**Figure 8:**
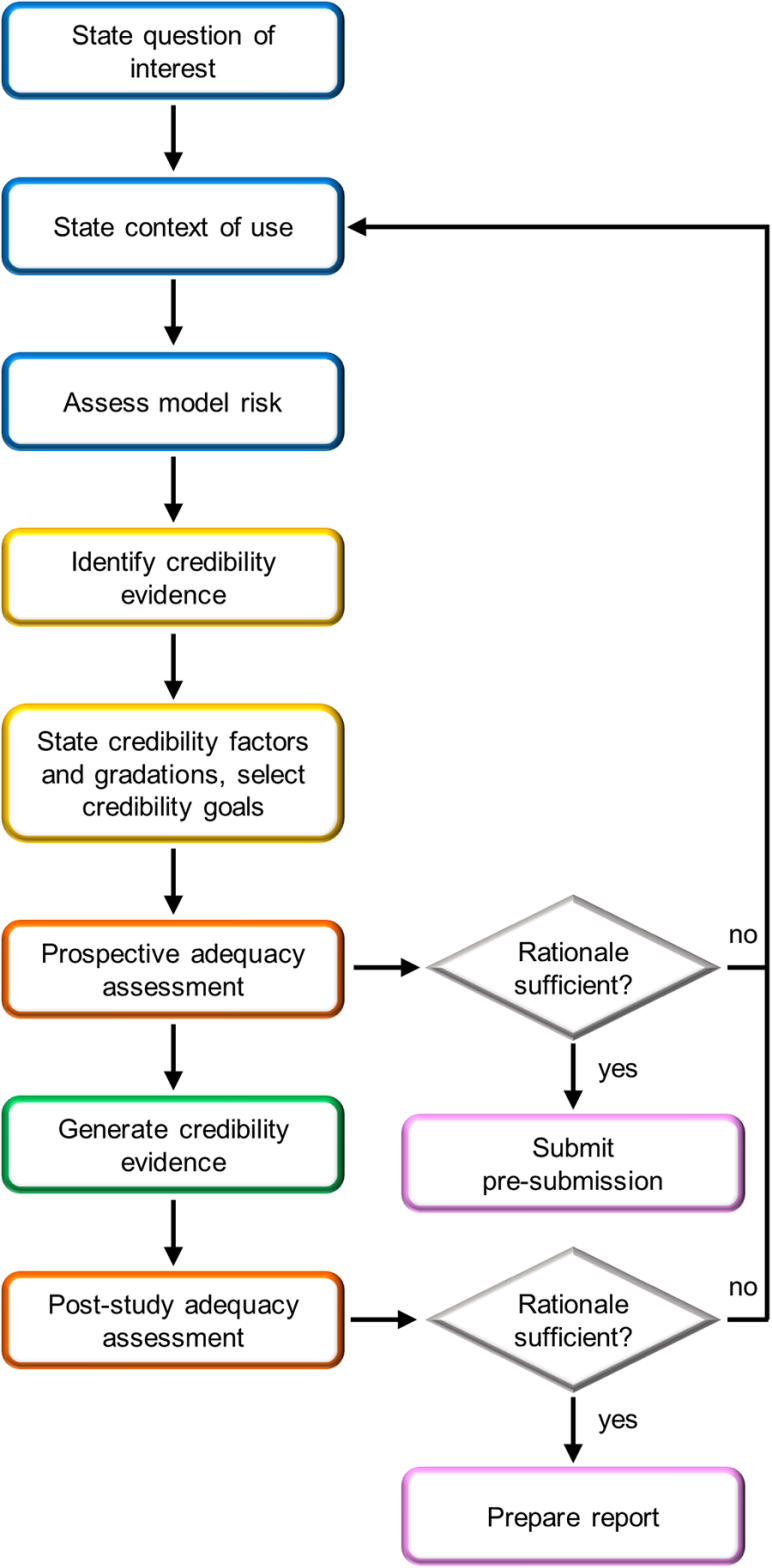
FDA risk-informed framework for the assessment of CM&S credibility in medical device regulatory submissions (11). Blue boxes are initial steps, yellow boxes are credibility assessment planning steps, orange boxes are adequacy assessment steps, pink boxes are steps related to FDA interaction, and the green box is study execution.

CM&S credibility assessment is essential to demonstrate the reliability of CM&S results for consideration in the medical device regulatory process. In this context, the ASME V&V 40–2018 standard proposes a risk-based credibility assessment framework for computational models intended for medical devices (12). This standard has been recently extended by the FDA in a guidance (11) supporting the integration of variedly validated CM&S data in medical device regulatory submissions. The framework is based on the principle that the context of use (COU) of the computational model determines the credibility requirements that must be met, so that higher risks associated to the use of the model lead to stricter requirements. Both the ASME standard (12) and the FDA guidance (11) provide general guidelines on planning and conducting VVUQ activities to assess whether credibility requirements are met. The various steps of the credibility assessment framework outlined in the FDA guidance (11) are reported in Figure 8. This section of the article aims to summarize the key aspects of the proposed credibility assessment framework, elucidating how the steps of the framework can be applied to FSI simulations of BMAVs intended to replace the preclinical *in vitro* tests recommended by ISO 5840:2021.

#### 3.3.1 Initial steps

**Figure 9:**
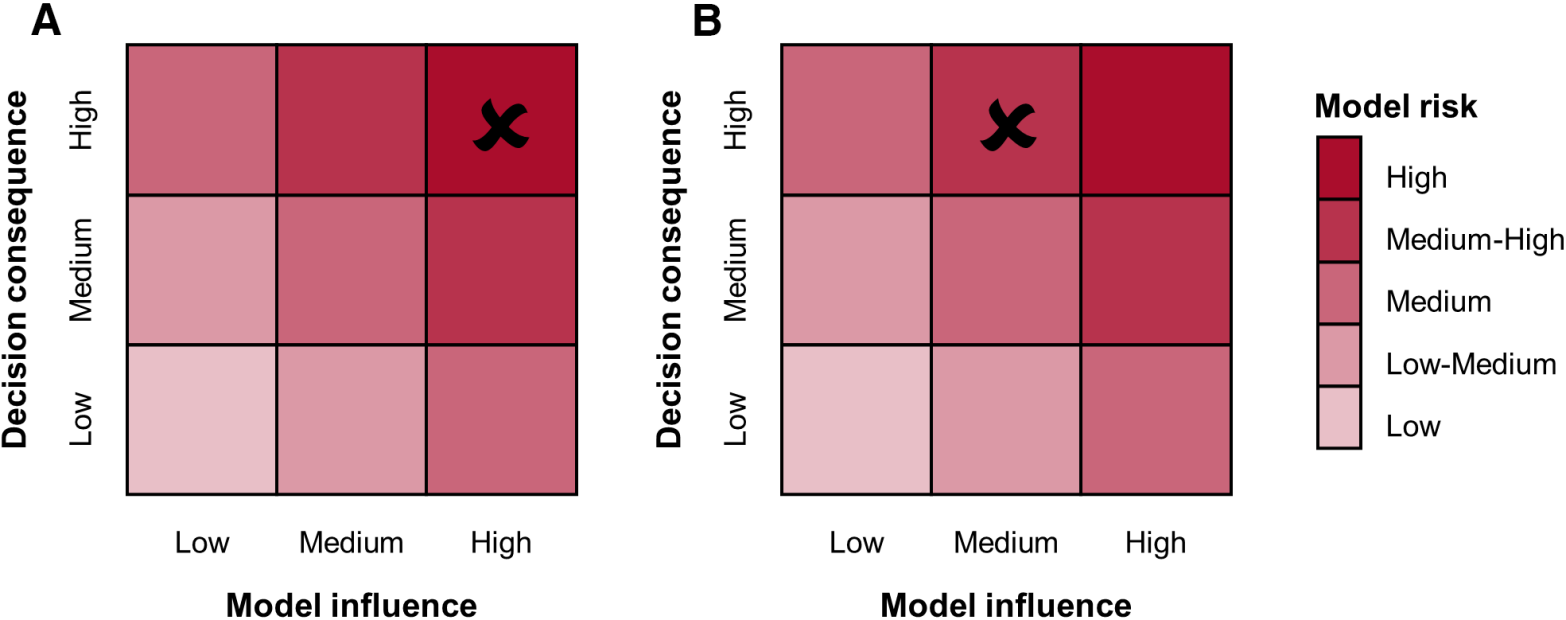
Model risk assessment schemes for FSI simulations of BMAVs intended for (**A**) replacing *in vitro* tests for the preclinical evaluation of minimum hydrodynamic performance and (**B**) assessing the blood damage potential through a combination of computational and experimental tests.

The credibility assessment process begins with the definition of a question of interest (QOI), which is the question to be addressed through the computational model. For example, in the case of a simulation intended to substitute the experimental pulsatile hydrodynamic minimum performance assessment of a BMAV, the QOI may be “Does the BMAV ensure an acceptable value of EOA?”. Based on the QOI, the COU of the model is defined. The COU describes how the QOI is addressed using the computational model. This also includes specifying the extent to which the results of the computational model will be supplemented by other sources of evidence. Therefore, the COU of FSI simulations aimed at assessing the thrombogenic and hemolytic potential of BMAVs will mention the integration of computational and experimental results. Conversely, in the case of hydrodynamic performance assessment, *in silico* tests may fully substitute other investigation approaches. This distinction also influences the definition of model risk, the next step of the credibility assessment framework. Model risk is defined as a combination of decision consequence and model influence. Decision consequence relates to the severity of adverse outcomes resulting from an incorrect answer to the QOI and is independent of the COU of the model. Conversely, model influence considers how strongly the answer to the QOI relies on model results compared to other sources of evidence. The incorrect assessment of both valve hydrodynamic performance and blood damage is associated to severe consequences for the patient, possibly including an increased risk of thromboembolic events (28). To mitigate the overall model risk, reducing model influence could involve integrating *in silico* results with experimental investigations. As previously mentioned, such integration is strongly recommended by ISO 5840:2021 for assessing the blood damage potential of BMAVs. In contrast, the integration of *in silico* hydrodynamic tests with *in vitro* tests may vary depending on the COU of the model. Figure 9 presents two illustrative schemes designed to evaluate model risk for BMAV FSI simulations. The first scenario involves simulations FSI intended to substitute *in vitro* tests in evaluating whether minimum hydrodynamic performance requirements are met. In this case, the high decision consequence is combined with high model influence since no other source of evidence are considered. Therefore, the resulting model risk is high. The second scenario pertains to FSI simulations intended to evaluate BMAV mechanically-induced blood damage potential. Although simulations may replace experimental flow field assessments, computational results will be integrated with *ex vivo* and preclinical *in vivo* data. Therefore, the model influence is medium and the resulting model risk is medium-high.

#### 3.3.2 Credibility evidence

After model risk assessment, the available or planned credibility evidence is identified and categorized. In this regard, the FDA guidance (11) defines eight credibility categories, which collect the results of different VVUQ activities. In general, VVUQ activities aim to establish trust in simulations (12). More specifically (i) verification activities address the software implementation of the simulation algorithm, (ii) validation focuses on comparing model results with those collected through a real-world comparator, typically consisting of *in vitro* or *in vivo* tests, and (iii) uncertainty quantification estimates the uncertainty in model outputs, particularly those arising from uncertainties in model inputs and uncertainties in model conceptual and mathematical formulation (11, 12). The FDA guidance suggests providing evidence for at least code verification, calculation verification and validation. In the specific case of simulations intended for *in silico* testing of medical devices, validation against bench test results should be considered. In the context of bench test validation activities, the FDA guidance defines three common scenarios: (i) prospectively planned validation, (ii) validation against retrospective results and (iii) the use of previously generated validation results. In prospectively planned validation, both simulations and comparator data are prospectively planned, thereby maximizing the relevance of the validation to the context of use, i.e., the applicability (11). Conversely, in validation against retrospective results, simulations are planned to enable the comparison with previously generated comparator data. Lastly, simulations and comparator data may have been previously generated and utilized to generate validation results available e.g., in regulatory submissions or in the literature.

Concerning FSI simulations of BMAVs, to the best of the authors’ knowledge, there are no examples in the literature of risk-based credibility assessment conducted in accordance with FDA and ASME guidelines (11, 12). Furthermore, any potential efforts made directly by BMAV manufacturers in this direction are not known. Nevertheless, verification and validation activities have been conducted in the past to increase the robustness and reliability of simulations results, as reported in Table 4. Code verification activities were typically undertaken when in-house solvers were adopted. While these activities are sometimes reported in works focusing on FSI simulations of BMAVs, readers are often directed to previous works specifically aimed at presenting, verifying, and validating the solver.

**Table 4:** Sensitivity analysis and validation activities conducted in FSI studies of BMAVs.

First Author, year (reference)	Sensitivity analysis	Quantitative validation	Comparator
Nowak, 2023 (34)	Mesh, time step	–	–
Nitti, 2022 (31)	Mesh	Flow velocity	Dasi et al. (76)
Ahmed, 2022 (36)	Mesh, time step	Flow velocity, platelet activation state	De Tullio et al. (67)
Sadipour, 2020 (38)	Mesh	Leaflet angular position	Dasi et al. (76)
Abbas, 2020 (40)	Mesh, time step	Leaflet angular position, flow velocity	Dumont et al. (75), Annerel et al. (48)
Abbas, 2020 (39)	Mesh	Leaflet angular position	Dumont et al. (75)
Yeh, 2019 (41)	Mesh	Flow velocity	Own
Banks, 2018 (43)	Mesh	–	–
Mirkhani, 2016 (45)	Mesh	Leaflet angular position	Dasi et al. (76)
Annerel, 2014 (48)	–	Leaflet angular position, flow velocity	Own
Li, 2012 (51)	–	Flow velocity	De Tullio et al. (67)
Kim, 2012 (52)	Mesh	Transvalvular pressure drop	Nobili et al. (33)
Annerel, 2012 (54)	Mesh, time step	–	–
Annerel, 2012 (55)	Algorithm parameters	–	–
Annerel, 2012 (56)	Mesh, time step, algorithm parameters	–	–
Hong, 2011 (57)	–	Leaflet angular position	Nobili et al. (33)
Hong, 2009 (65)	–	Leaflet angular position, transvalvular pressure drop	Nobili et al. (33)
Guivier-Curien, 2009 (66)	–	Leaflet angular position, flow velocity	Own
De Tullio, 2009 (67)	Mesh	Leaflet angular position, flow velocity	Cerroni (105)
Choi, 2009 (68)	Mesh	Leaflet angular position, flow rate	Guivier et al. (73)
Nobili, 2008 (33)	–	Leaflet angular position, transvalvular pressure drop	Own
Borazjani, 2008 (69)	Mesh	Leaflet angular position, vorticity	Dasi et al. (76)
Tai, 2007 (70)	Mesh	–	–
Palmieri, 2007 (71)	–	Flow velocity	Own
Nobili, 2007 (72)	–	Leaflet velocity and displacement, flow rate	Own
Guivier, 2007 (73)	Mesh, time step	–	–
Dasi, 2007 (76)	–	Vorticity	Own
Bang, 2006 (78)	Mesh, time step	–	–
Bang, 2005 (80)	Mesh, time step	–	–
Redaelli, 2004 (81)	–	Leaflet velocity and displacement, flow rate	Own
Choi, 2001 (85)	Mesh	Flow velocity	King et al. (106)

Calculation verification activities were conducted by analyzing result sensitivity to discretization grid (31, 34, 36, 38–41, 43, 45, 52, 54, 56, 67–70, 73, 78, 80, 85), to time step (34, 36, 40, 54, 56, 73, 78, 80) and specific parameters of the FSI algorithm (55, 56), as reported in Table 4.

Validation of simulation results has been pursued through various approaches. For instance, prospectively planned validation activities were conducted in studies where both experimental and computational data were collected and compared, with FSI simulation reproducing the experimental set-up (33, 41, 48, 66, 71, 72, 76, 81) (Table 4). Prospectively planned validation is feasible solely when in-house experimental tests can be conducted. Therefore, validation was often conducted against retrospective data either by qualitatively highlighting similarities in results (34, 44, 47, 49, 50, 55, 56, 58–62, 64, 73, 75, 78–80, 82, 83) or, more rigorously (Table 4), through a quantitative comparison of simulations and experimental results (31, 36, 38–40, 45, 51, 52, 57, 65, 67–69, 85).

#### 3.3.3 Credibility factors and credibility goals

ASME V&V 40–2018 (12) provides a list of suggested credibility factors concerning the various aspects of verification and validation activities as well as applicability assessment. For each credibility factor, a gradation of activities should be defined, accounting for different levels of investigation rigor. Then, the proper credibility goal should be selected from the gradation, commensurate with model risk. Credibility factors may also be addressed through previously generated data. In this case, the gradation level of the activities conducted to collect these data should be identified. If the credibility goals selected for planned activities or the credibility level of previously conducted activities do not align with the model risk, the adequacy of the activities should be appropriately justified. The gradation associated with credibility factors of verification and validation activities should be determined based on the specific COU of the simulation and the expertise of the investigators. However, in this context, considering the medium-high and high model risk levels previously discussed for FSI simulations intended to replace *in vitro* BMAV biomechanical assessment (Section 3.2), it is advisable to select credibility goals corresponding to high rigor in the gradation of credibility assessment activities.

#### 3.3.4 Adequacy assessment

The adequacy assessment evaluates the strength of the credibility assessment in supporting the use of the computational model for the intended COU (11). The post-study adequacy assessment is based on assessing whether the credibility goals were achieved and establishing whether the collected evidence is strong enough to justify the use of the computational model (11). If the evidence collected is deemed insufficient, it may be necessary to consider model modifications and further evidence collection. In addition to the post-study evaluation, an adequacy assessment can also be performed prospectively, particularly when seeking a feedback from the FDA on the planned credibility assessment activities. Considering FSI simulations of BMAVs, a prospective adequacy assessment may be advisable, because prospectively planned validation activities may require consistent economic resources for the generation of comparator data. Therefore, if prospectively planned validation is being considered, a prospective adequacy assessment can play a crucial role in minimizing unnecessary costs.

## 4 Conclusions

The present narrative review discusses the state of the art of FSI simulations of BMAVs and their potential to complement and partially replace *in vitro* testing in the preclinical and post-market phases of device development, in accordance with FDA and ASME guidelines (11, 12). The FSI simulation approach enables a detailed investigation of BMAV fluid dynamics, as well as the potential adverse biological events determined by the implantation of these devices. The literature analysis presented the main features of available FSI models of BMAVs in terms of aortic and valve model, rheological and turbulence models, boundary conditions, FSI algorithms and applications of the model. The use of FSI simulations can be further extended by conducting simulations to complement experimental tests throughout the total product life cycle of BMAVs. Specifically, in the preclinical device evaluation phase and in the post-market assessment phase, FSI simulations can replicate bench tests, thus partially replacing *in vitro* experimentation. The evidence collected through BMAVs *in silico* testing can also support devices regulatory submission, provided that simulation credibility is assessed through VVUQ activities accounting for the simulation COU.

Although the studies based on FSI analysis conducted so far have covered many aspects of investigation for BMAVs, there are still some biomechanical aspects that have not been explored. For instance, the effect of valve implantation height relative to the aortic annulus has not been investigated with FSI simulations. Moreover, coronary arteries were rarely included in aortic geometrical models. Therefore, it would be advisable to further investigate how the presence of both coronary arteries and aortic sinuses impacts aortic root fluid dynamics past a BMAV. Additionally, only a few FSI studies compared the fluid dynamics of different mechanical aortic valves, and none of them accounted for aortic curvature and deformability.

Regarding the use of FSI simulations for BMAV preclinical and post-market biomechanical evaluation, it is reasonable to expect that in the future BMAV manufacturers will increasingly rely on simulations to gather evidence supporting device development and regulatory submissions. In this regard, it will be essential to plan and justify the selection of simulation modelling strategies based on simulation COU, as recommended by FDA and ASME guidelines (11, 12).
